# An adaptable parallel algorithm for the direct numerical simulation of incompressible turbulent flows using a Fourier spectral/*hp* element method and MPI virtual topologies

**DOI:** 10.1016/j.cpc.2016.04.011

**Published:** 2016-09

**Authors:** A. Bolis, C.D. Cantwell, D. Moxey, D. Serson, S.J. Sherwin

**Affiliations:** Department of Aeronautics, Imperial College London, South Kensington Campus, London, UK

**Keywords:** Spectral/hp element method, High-order methods, Incompressible flows, MPI parallelisation, Virtual topologies

## Abstract

A hybrid parallelisation technique for distributed memory systems is investigated for a coupled Fourier-spectral/*hp* element discretisation of domains characterised by geometric homogeneity in one or more directions. The performance of the approach is mathematically modelled in terms of operation count and communication costs for identifying the most efficient parameter choices. The model is calibrated to target a specific hardware platform after which it is shown to accurately predict the performance in the hybrid regime. The method is applied to modelling turbulent flow using the incompressible Navier–Stokes equations in an axisymmetric pipe and square channel. The hybrid method extends the practical limitations of the discretisation, allowing greater parallelism and reduced wall times. Performance is shown to continue to scale when both parallelisation strategies are used.

## 1 Introduction

Direct Numerical Simulation (DNS) is used to simulate complex laminar and turbulent flow problems both to gain an understanding of the fundamental flow physics and for industrial applications [1]. Turbulent flows inherently require high spatial and temporal resolutions in order to resolve the spectrum of scales within the flow, which depends on the Reynolds number Re. The number of grid points needed to resolve a fully turbulent three-dimensional flow scales as $Re^{9/4}$   [2], meaning that even at modest Re, the computational demands are significant. Since practical CFD applications involve Reynolds numbers on the order of $10^{3}\text{–}10^{6}$ and higher, this inevitably makes serial computation impossible, necessitating the use of parallel clusters of computers.

The most prevalent form of high-performance computer systems are distributed-memory clusters consisting of an interconnected collection of processors, each with their own local memory hierarchies. Traditionally, the capacity of these systems has been broadly increased through faster processor clock speeds and improved lower-latency network interconnects. However, in recent years, HPC facilities have evolved around increasingly parallel systems as clock speeds have saturated and energy-usage concerns become a motivating factor  [3]. Consequently, algorithms have been required to adapt to the changing hardware landscape in order to maintain efficiency.

The spectral/*hp* element method  [4], whilst being used to simulate fluid flow for many years in an academic setting, is now emerging as an attractive alternative to many traditional numerical discretisations on modern HPC hardware. As opposed to the classical finite element method, spectral/*hp* elements use high-order polynomial expansions on each element. Numerically, this has the advantage of low dispersion and diffusion alongside exponential convergence in the polynomial order. Additionally however, discretised operators are dense and have a far richer structure compared to linear expansions, meaning that they can more effectively utilise caching on modern HPC hardware. The tensor product of one-dimensional basis functions on each element also admits a rich fabric of implementation strategies  [5], [6], [7].

However, variations of this method mean that we can further improve computational performance whilst preserving the accuracy of the simulation. Many studies of fundamental flow physics are posed on domains which are characterised by geometric homogeneity in one or more coordinate directions [8], [9], [10]. Instead of discretising the domain using a 3D spectral/*hp* element method, one can combine a 2D spectral/*hp* element discretisation with a pure spectral expansion to significantly reduce the computational cost of these problems. This approach is known as a *Fourier–spectral/*hp* element method*   [11]. In this study we are specifically interested in the case where only one coordinate direction possesses geometric homogeneity. Therefore, the 3D domain is decomposed into a sequence of spectral/*hp* element planes, coupled using a Fourier expansion in the third coordinate directions.

The approach typically used when parallelising this type of discretisation is to either use mesh-decomposition in the spectral/*hp* element planes, or apply a modal decomposition in the Fourier direction. The latter takes advantage of the orthogonality property of the Fourier basis for linear operators. The optimal choice of parallelisation strategy typically depends on the size of the problem, the ratio of Fourier planes to spectral elements, alongside the hardware and interconnect of the parallel system. Moreover, the fast development of computer systems forces software designers to make a continuous effort to maintain algorithms to be able to exploit all the benefits exposed by the latest generation of hardware. There is therefore benefit to be gained from a code supporting both types of parallelism, but predicting the performance of these algorithms on a specific architecture is not trivial  [12].

The performance of a parallel 3D incompressible Navier–Stokes solver using the Fourier–spectral/*hp* element method has been benchmarked previously [13], [14]. Spectral modes were distributed across the processes, requiring the transposition of data using the MPI all-to-all technique to compute derivatives in that direction. Their performance model assumed a flat communication topology and the maximum number of processes was limited to the number of Fourier planes. Conversely, parallelisation of a spectral element discretisation has been explored [15], [16], [17], [18], [19], for which the upper limit on the number of processes is the number of mesh elements.

Performance of a mixed-parallelism case for 3D turbulence simulations has previously been investigated  [20], specifically for a 1D spectral/*hp* element discretisation, coupled with a 2D spectral expansion. Parallel communication was implemented across processes as a Cartesian topology and a performance model was constructed which suggested improved strong scaling could be achieved on specific architectures. Solver performance depends on hardware characteristics such as memory bandwidth and processor cache size, but also on network capabilities in terms of latency, and bandwidth. Therefore prudent choice of parallelism strategy can enable improved overall performance by structuring the computation and communication pattern to better match the available hardware.

The present study is distinguished from this previous work by the choice of discretisation. We use a two-dimensional spectral/*hp* element method, coupled with a one-dimensional spectral expansion. This permits the investigation of flow problems on geometries of significantly greater complexity than earlier works. We first outline the discretisation and parallelisation strategies and quantify their comparative performance. For large runs with many processes the number of possible hybrid parallelism strategies may be significant. We construct a mathematical model to characterise the expected performance of any given single or hybrid parallelisation strategy which can be used to predict the optimal strategy for a given problem. We calibrate the model against the individual mesh-decomposition and Fourier parallelisation techniques and demonstrate its accuracy in predicting performance of the hybrid approach.

## 2 Methods

Three-dimensional incompressible, isothermal flow with constant density and viscosity is governed by the incompressible Navier–Stokes equations which, in terms of the primitive variables (u,p), are expressed as

$$\frac{\partial u}{\partial t}+u\cdot \nabla u=-\nabla p+\nu \nabla^{2}u,\nabla \cdot u=0\text{,}$$

where p is the kinematic pressure, ν is the kinematic viscosity and $u={\left[u,v,w\right]}^{⊤}$ is the velocity.

### 2.1 Spatial discretisation

**Fig. 1:**
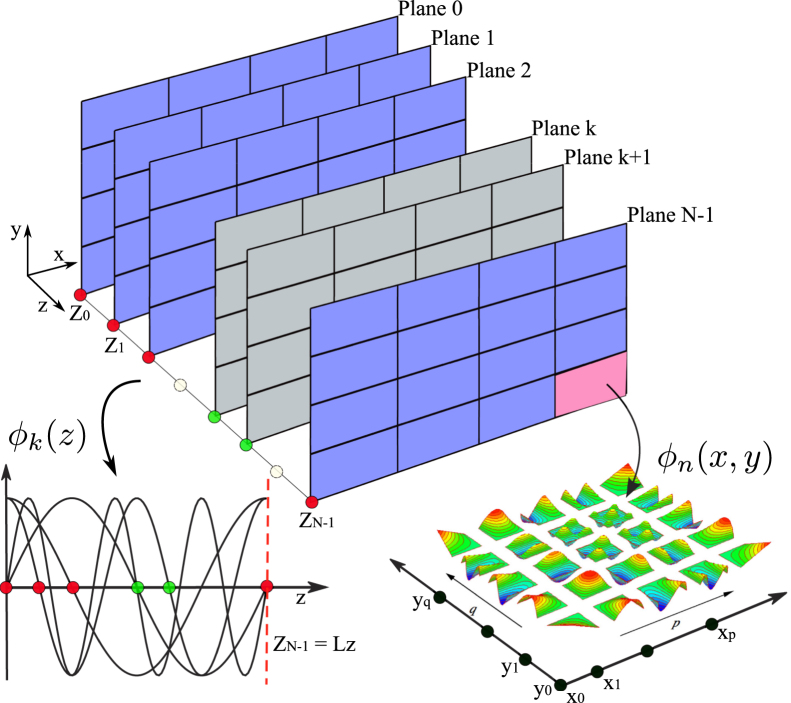
Structure of a three-dimensional expansion using a Fourier spectral/*hp* element method.

The three-dimensional domain is decomposed into $N_{Z}$ two-dimensional spectral/*hp* element planes spanning the x and y coordinate directions, coupled with a Fourier expansion in the third homogeneous direction, as illustrated in Fig. 1. The spectral/*hp* element discretisation is described elsewhere  [4] and only a brief summary is given here.

Each two-dimensional plane $\Omega_{k}$ is partitioned into a set of $N_{\mathrm{el}}$ subdomains $\Omega_{k}^{e}$ such that,

$$\Omega_{k}=\overset{N_{\mathrm{el}}}{\underset{e=0}{⋃}}\Omega_{k}^{e}\Omega_{k}^{e}\cap \Omega_{k}^{f}=0̸\;\forall e\neq f\text{.}$$

In this study the meshes consist of quadrilateral elements only, but the approach may be equally applied when using triangular elements. Numerical integration and differential operators are constructed on a standard reference element $\Omega^{\mathrm{st}}$ which is mapped to each $\Omega_{k}^{e}$ using a bijective map, $\chi^{e}:\Omega^{\mathrm{st}}\to \Omega^{e}$, as $x=\chi^{e}\left(\xi \right)$. On each element, the solution u may be approximated as

$$u^{\delta }\left(x,y\right)=\overset{N}{\underset{n=0}{\sum }}\phi_{n}\left(x,y\right){\overset{ˆ}{u}}_{n}=\overset{P}{\underset{p=0}{\sum }}\overset{P}{\underset{q=0}{\sum }}\phi_{p}\left(x\right)\phi_{q}\left(y\right){\overset{ˆ}{u}}_{pq}^{e}\text{,}$$

where ${\overset{ˆ}{u}}_{pq}^{e}$ are elemental coefficients. These correspond to the tensor-product of nodal expansion bases, $\phi_{p}\left(x\right)$ and $\phi_{q}\left(y\right)$, of order P defined as Lagrange polynomials through Gauss–Lobatto–Legendre points $\xi_{i}$, and have the form

$$\phi_{m}\left(\xi \right)=\frac{\overset{P}{\underset{l=0,l\neq m}{\prod }}\left(\xi -\xi_{l}\right)}{\overset{P}{\underset{l=0,l\neq m}{\prod }}\left(\xi_{m}-\xi_{l}\right)}\text{.}$$

This is synonymous with the original spectral element method, giving a total of ${\left(P+1\right)}^{2}$ degrees of freedom (DOF) per element and $N_{XY}=N_{el}\times {\left(P+1\right)}^{2}$ local degrees of freedom per plane. Gaussian quadrature is used for numerical integration, for which the solution u is represented on the same set of P+1 points $\xi_{m}$.

The connectivity of elements in a plane is represented by an assembly mapping A which maps the concatenated vector of elemental degrees of freedom to their global counterparts and enforces a $C^{0}$-continuity constraint. The global degrees of freedom are assembled using the relation ${\overset{ˆ}{u}}^{e}=A{\overset{ˆ}{u}}^{g}$, where A is the matrix equivalent of A. This matrix is in general highly sparse and so is in practice not constructed explicitly.

Operators in the spectral/hp element method are constructed elementally and applied using the sum-factorisation technique  [21] as this has been demonstrated to be more efficient when operating on elements with higher-order bases [7], [6], [5]. The tensor-product nature of the elemental expansion bases allows matrix–vector operations to be decomposed into a sequence of smaller, more computationally efficient matrix–matrix operations, performed in each coordinate direction separately.

In the z-direction, the solution is expressed using a Fourier basis of $N_{Z}/2$ complex modes, $\phi_{k}\left(z\right)=e^{izk}$, to give an expansion of the three-dimensional solution on an element as

$$u^{\delta }\left(x,y,z\right)=\underset{n}{\sum }\phi_{n}\left(x,y,z\right){\overset{ˆ}{u}}_{n}=\underset{pqk}{\sum }\phi_{pq}\left(x,y\right)\phi_{k}\left(z\right){\overset{ˆ}{u}}_{pqk}\text{.}$$

The total number of degrees of freedom is therefore $N_{\mathrm{tot}}=N_{XY}N_{Z}$.

### 2.2 Temporal discretisation

A stiffly stable splitting scheme  [22] is adopted which decouples the velocity and pressure fields, leading to an explicit treatment of the advection term and an implicit treatment of the pressure and the diffusion terms. The key steps are

$$\frac{\overset{¯}{u}-\overset{J-1}{\underset{q=0}{\sum }}\alpha_{q}u^{n-q}}{\Delta t}=-\overset{J-1}{\underset{q=0}{\sum }}\beta_{q}{\left[\left(u\cdot \nabla \right)u\right]}^{n-q},\nabla^{2}p^{n+1}=\nabla \cdot \left(\frac{\overset{¯}{u}}{\Delta t}\right),\frac{\overset{¯}{\overset{¯}{u}}-\overset{¯}{u}}{\Delta t}=-\nabla p^{n+1},\frac{\gamma_{0}u^{n+1}-\overset{¯}{\overset{¯}{u}}}{\Delta t}=\nu \nabla^{2}u^{n+1}\text{.}$$

To maintain the order of the scheme, a modified Neumann pressure boundary condition is used,

$${\frac{\partial p}{\partial n}}^{n+1}=-\left[{\frac{\partial u}{\partial t}}^{n+1}+\nu \overset{J-1}{\underset{q=0}{\sum }}\beta_{q}{\left(\nabla \times \omega \right)}^{n-q}\;+\overset{J-1}{\underset{q=0}{\sum }}\beta_{q}{\left[\left(u\cdot \nabla \right)u\right]}^{n-q}\right]\cdot n\text{.}$$

**Fig. 2:**
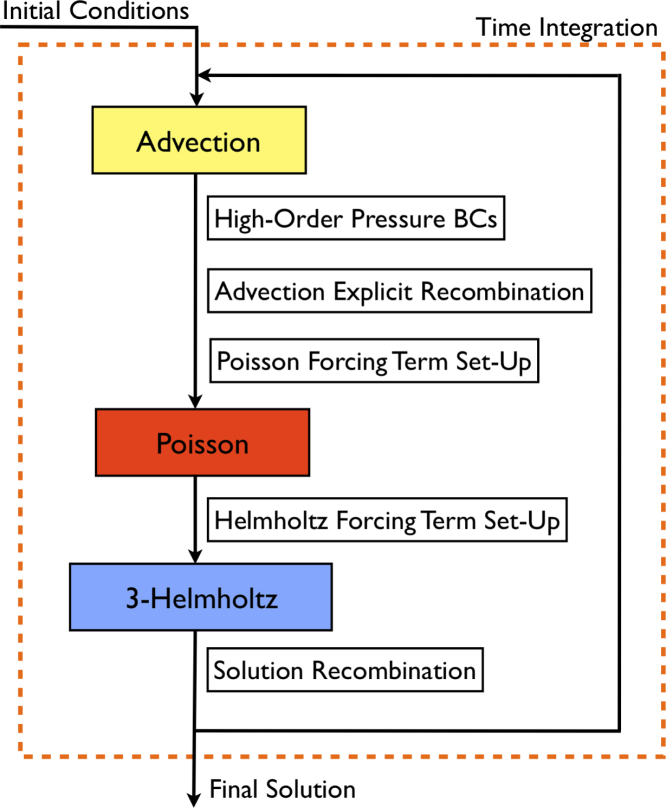
Incompressible Navier–Stokes solution algorithm. Details of the building blocks of the time-integration process. The most expensive routines are highlighted, i.e. the advection term calculation and the elliptic solvers for pressure and velocity (Poisson and Helmholtz).

The coefficients $\alpha_{q}$, $\beta_{q}$ and $\gamma_{0}$ can be found in  [22] for first-, second- and third-order implicit–explicit (IMEX) time-integration schemes. Fig. 2 illustrates the implementation of the time-integration section of the algorithm, where we ignore input/output and set-up costs. For short time-integration, these may be significant.

### 2.3 Parallelisation

In this section we describe the parallelisation approaches used in this study. We provide an overview of the two orthogonal approaches: parallel decomposition of the Fourier modes (modal parallelisation) and parallel decomposition of the spectral/*hp* element planes (elemental parallelisation). We then outline the hybrid approach which combines both techniques, and describe its implementation. In each case we partition the simulation across a total of R processes. The Message Passing Interface (MPI) library is used for communication in all three methods.

In modal parallelisation the $N_{Z}$ planes, corresponding to $N_{Z}/2$ complex Fourier modes, are distributed equally among the processes. Elliptic solves are decoupled in the Fourier-transformed space and can be performed independently on each plane using either a direct Cholesky factorisation with reverse Cuthill–McKee algorithm (LAPACK)  [23], or through an iterative conjugate gradient algorithm. The non-linear advection term is more efficiently computed in non-modal space. To perform the inverse and forward Fourier transforms, used before and after the advection calculation respectively, the data to be transformed must reside on the same process. In practice, this requires a transposition of the data using an MPI all-to-all operation. To support efficient differentiation in the z-coordinate direction, we additionally impose the constraint that both the real and imaginary components of each complex Fourier mode reside on the same process, since, in the Fourier space, derivatives are calculated through the multiplication ${\overset{ˆ}{u}}_{k}\mapsto -ik{\overset{ˆ}{u}}_{k}$. This restricts the maximum number of useable processes to $N_{Z}/2$.

In contrast, elemental parallelism distributes the $N_{\mathrm{el}}$ elements of each plane among the processes. The partitioning of the 2D plane is implemented using the METIS graph partitioning library  [24] and an identical partitioning and distribution amongst processes is used for each plane in the domain. The natural limit on the number of useable processes is therefore $N_{el}$. The dual-graph of the mesh is partitioned among the R processes to equally distribute the number of degrees of freedom, whilst minimising the edge-cut, and therefore the inter-process communication. Elliptic solves are performed iteratively, with communication being required to exchange boundary information between adjacent elements residing on different processes at each iteration. This data exchange is implemented using the gather–scatter algorithm from Nek5000  [25] which uses a global numbering of the DOFs in the plane to efficiently summate process-local contributions and distribute the result back to the participating processes.

**Fig. 3:**
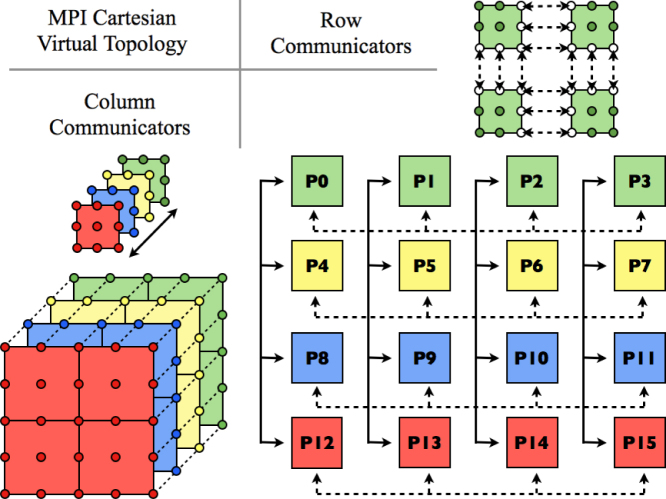
Illustrative MPI Cartesian communicator for a hybrid parallelisation of a Fourier spectral/*hp* element discretisation using 4 elements per plane and 4 planes, on 16 MPI processes. Row communicators handle the communication between mesh partitions for elemental parallelisation while column communicators handle communication between planes for modal parallelisation.

Hybrid parallelisation combines both modal and elemental approaches by organising the available processes in a Cartesian grid  [20], as illustrated in Fig. 3. In this arrangement, the world communicator is split into a series of row communicators which support elemental parallelisation, while column communicators enable modal parallelisation. Each process belongs to precisely one row communicator and one column communicator and nominally operates on a fixed subset of elements in a fixed subset of planes. As in modal parallelism, elliptic solves are performed in Fourier-transformed space, but due to the elemental parallelism the iterative conjugate gradient solver must be used. The limit on the number of viable processes is now increased substantially to $N_{el}\times N_{Z}/2$.

### 2.4 Test environment

All simulations are performed on an SGI Altix ICE 8200 EX system with up to 512 cores (64 eight-core nodes). Each node contains Nehalem CPUs running at 2.93 GHz and 24 GB RAM. Communication is through a dual-rail *Infiniband* interconnect. The system runs Redhat Enterprise Linux with kernel version 3.0.58-0.6.6. Intel MPI was used for parallel message exchange and FFTW 3.2.2 for performing fast Fourier transforms.

The software used for the spectral/hp element discretisation in this study was Nektar++ v3.3.0  [26], [27]. In summary, the framework provides scope for constructing high-order polynomial expansions on both fully two-dimensional and three-dimensional domains. It also supports a coupled spectral/hp element—Fourier approach for domains with geometric homogeneity. The specific operators and time integration necessary for solving the incompressible Navier–Stokes equations are built upon this framework. As with any numerical timing study, the presented results are specific to the implementation used, although should provide useful generic guidance.

## 3 Performance model

Identifying the optimal strategy and the distribution of processes between elemental and modal parallelism is non-trivial, since algorithmic complexity and specific system architecture affects performance. We therefore design a performance model, calibrated through the use of the two parallelisation strategies independently, to help select the best approach before the start of a given simulation.

### 3.1 General model assumptions

To ensure the model remains simple enough for predictive use, yet sufficiently complex to provide reasonable accuracy, we make a number of assumptions regarding the nature of the computational problem and hardware when evaluating the computational and communication costs.

The computational cost of an algorithmic unit is evaluated using the floating-point operation count of basic routines such as matrix–vector multiplications, inner products and vector–vector summations. This implicitly disregards hardware characteristics, such as caching and memory throughput limits, although our testing has shown that these aspects can be reasonably captured using scalar constants, determined during the calibration process for a specific platform. Operations are evaluated at the element level, and their total computational cost across the domain is therefore assumed to be predominantly independent of the parallelisation strategy.

Communication costs are generally more complex to model and strongly depend on the hardware configuration. Different cluster configurations, such as mesh, hypercube or ring interconnect topologies, have a significant effect on the measured communication time. In this study we follow the most common approach when estimating communication costs  [20], [14], which is to assume a “flat” topology supporting direct communication between nodes and no interconnect contention. Operations are assumed to be performed using double-precision floating point numbers, occupying eight bytes on the test system described above.

### 3.2 Model construction

The general structure of our performance model is as follows. Let $O_{i}$ be the operation count of the ith operation in the algorithm. Let $C_{j}$ be the time required for the jth communication, then we can define the total parallel execution time, T, as

$$T=\frac{1}{R_{XY}R_{Z}}\underset{i}{\sum }O_{i}+\underset{j}{\sum }C_{j}$$

where $R_{XY}$ and $R_{Z}$ are the number of processes used for elemental and Fourier parallelism, respectively, and $R=R_{XY}R_{Z}$. The size of a computational problem is generally measured by the number of degrees of freedom. In the spectral/*hp* discretisation, this corresponds to the number of elemental modes which, for two-dimensional quadrilateral elements, is ${\left(P+1\right)}^{2}$. For some matrix–vector operations the sum-factorisation technique, which exploits the tensorial nature of the expansion, can be used that requires $\left(4P^{3}+18P^{2}+26P+12\right)$ operations per element  [7].

The communication times $T_{Cj}$ can be further modelled as

(1) $$C_{j}=N_{\mathrm{msgs}}\times \left[\tau_{L}+N_{\mathrm{op}}\times \tau_{B}\right]$$

where $N_{\mathrm{msgs}}$ is the number of messages transmitted during an operation, $N_{\mathrm{op}}$ is the number of floating-point values per message, $\tau_{L}$ is the latency and $\tau_{B}$ the inverse of the bandwidth. Note that $\tau_{B}$ is quantified using s/DOF, rather than the conventional s/MB, to facilitate the modelling. Latency and bandwidth are sampled for the test system using the MPI benchmarking application IBM-MP1. Bandwidth is measured for a number of MPI routines and for messages of size 8 bytes up to 4 MB and averaged. For the test system considered, the average bandwidth measured was $1.64\cdot 10^{3}\;\mathrm{MB}/s$. Bandwidth and latency for the test system was determined to be

$$\tau_{B}=4.87\cdot 10^{-9}\;s/\mathrm{DOF}\text{,}\;\tau_{L}=2.09\cdot 10^{-6}\;s\text{.}$$

### 3.3 Advection term

We first model the advection term u⋅∇u, which is computed in physical space and can be expanded as

N(u)=u∂u/∂x+v∂u/∂y+w∂u/∂z,N(v)=u∂v/∂x+v∂v/∂y+w∂v/∂z,N(w)=u∂w/∂x+v∂w/∂y+w∂w/∂z,

where u, v and w denote the three components of the velocity u. The numerical implementation of this is shown in Algorithm 1. Computational costs arise from FFTs (lines 1, 4 and 6), derivatives (lines 2 and 3) and vector–vector operations (line 5), while communication is only required to compute the FFTs.

**Figure:**
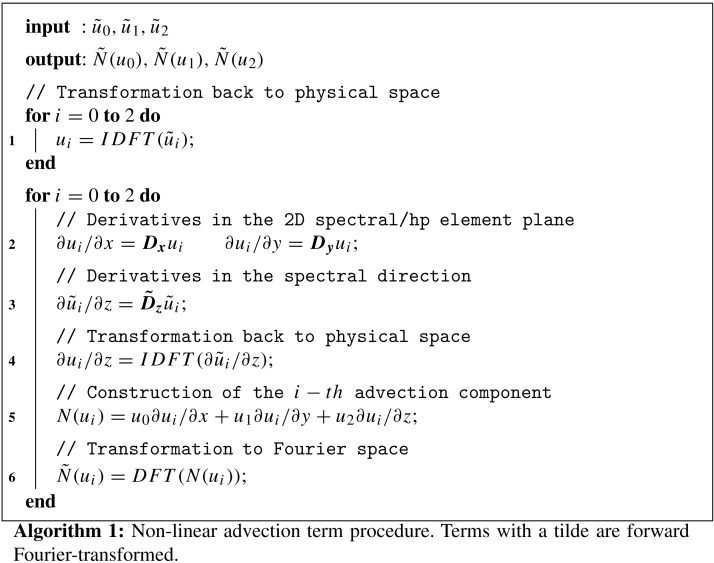

Inverse FFTs are required for each of the velocity components and the z-derivatives of each of the velocity components. A forward FFT is used to transform the result of the advection calculation. This gives a total of nine FFTs, each consisting of a set of independent 1D FFTs. The number of 1D FFTs is given by the number of quadrature points associated with the local spectral/*hp* element mesh partition. Assuming the mesh is evenly partitioned, we can quantify the total number of 1D FFTs as $N_{el}{\left(P+1\right)}^{2}$, each costing $O\left(N_{Z}\log_{2}\left(N_{Z}\right)\right)$, giving a cumulative cost of

$$O_{1}^{A}=9N_{el}{\left(P+1\right)}^{2}\cdot C_{\mathrm{FFT}}N_{Z}\log_{2}N_{Z}\text{,}$$

where $C_{\mathrm{FFT}}$ is a const. Derivatives in the z-direction are a BLAS level 1 operation with total cost

$$O_{2}^{A}=N_{el}N_{Z}{\left(P+1\right)}^{2}\text{.}$$

In-plane physical derivatives will be proportional to the cost of executing a matrix–vector multiplication using the general derivative matrix. A total of six in-plane derivatives are computed. The total cost of derivative operations is therefore,

$$O_{3}^{A}=6N_{el}{\left(P+1\right)}^{4}\text{.}$$

Finally, there are five level 2 BLAS operations in calculating each component of $N\left(u_{i}\right)$. Vectors are of size $N_{el}{\left(P+1\right)}^{2}$, resulting in a total cost of

$$O_{4}^{A}=15N_{el}{\left(P+1\right)}^{2}\text{.}$$

For the advection term, communication is required during the 9 FFTs to shuffle data between processes so that the data for each 1D FFT, previously spanning $R_{Z}$ processes, is colocated on the same process. We apply the communication model described in (1). For each of the 9 FFTs two MPI All-to-all calls are required (shuffling and unshuffling), each of which formally requires $N_{\mathrm{msgs}}=\left(R_{Z}-1\right)$ messages [20], [14]. Message size is based on the assumption that the 1D FFTs will be evenly distributed across the participating processes. This gives a communication cost of

$$C_{1}^{A}=18\left(R_{Z}-1\right)\left(\tau_{L}+\frac{N_{el}}{R_{Z}R_{XY}}{\left(P+1\right)}^{2}\tau_{B}\right)\text{.}$$

Combining the above contributions and distributing the computational cost amongst the processes gives a parallel execution time of

$$T^{A}=\frac{1}{R_{Z}R_{XY}}\cdot \underset{i}{\sum }\overset{A}{\underset{i}{O}}+\underset{j}{\sum }\overset{A}{\underset{j}{C}}\text{.}$$

### 3.4 Elliptic solver

Algorithm 2 shows the basic steps to solve the linear systems using a preconditioned conjugate gradient method  [28]. To simplify the analysis, we do not perform static condensation of the elliptic systems, evaluating them using a block-diagonal matrix system, where each block contains a full elemental matrix.

**Figure:**
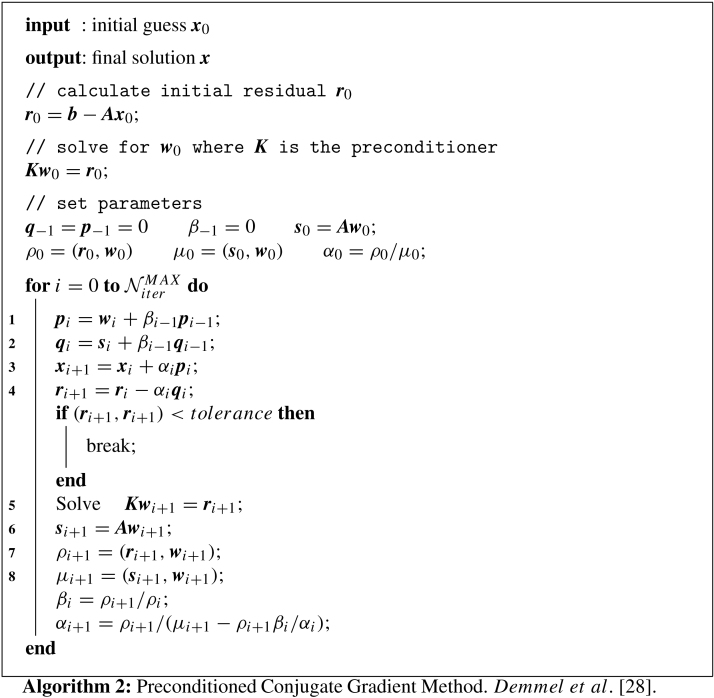

The daxpy operations on lines 1–4, each comprising one scalar–vector multiplication and one vector–vector summation giving a total cost of

$$O_{1}^{E}=8N_{el}{\left(P+1\right)}^{2}\text{.}$$

The application of the diagonal preconditioner in step 5 can be considered a vector–vector multiplication and has cost

$$O_{2}^{E}=N_{el}{\left(P+1\right)}^{2}\text{.}$$

The most computationally expensive step is the evaluation of the matrix system in step 6. Applying the sum-factorisation operation count defined earlier in this section we quantify the number of operations as

$$O_{3}^{E}=N_{el}\left(4P^{3}+18P^{2}+26P+12\right)\text{.}$$

Finally, the two inner products in steps 7 and 8 evaluate the stopping criteria of the iterative algorithm. Each consist of a vector–vector multiplication and a sum reduction. The vector–vector multiplication requires $N_{el}{\left(P+1\right)}^{2}$ operations per plane while the sum reduction $N_{el}{\left(P+1\right)}^{2}-1$ operations per plane. In order to maintain simplicity in the model we approximate the sum reduction to $N_{el}{\left(P+1\right)}^{2}$ operations, leading to

$$O_{4}^{E}=O_{8}^{E}=6N_{el}{\left(P+1\right)}^{2}\text{.}$$

Communication appears during the inner product reductions and during the matrix–vector multiplication. The inner product reduction can be modelled using the All-gather model  [20]. The number of messages is $\left(R_{XY}-1\right)$ for each inner product, since the local reductions need to be composed into a global reduction, which happens on one processor. Since the reduction operations for each iteration can be collated into a single message, the size is three floating-point values. This results in the communication time

$$C_{1}^{E}=\left(R_{XY}-1\right)\left(\tau_{L}+3\tau_{B}\right)\text{.}$$

**Fig. 4:**
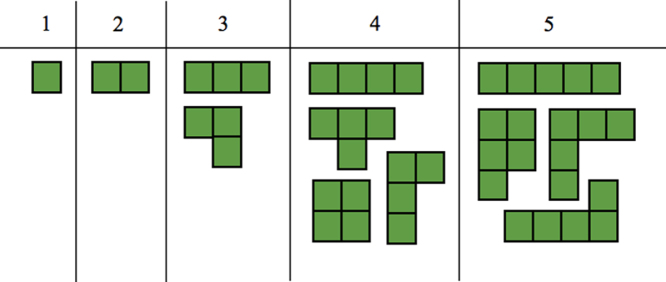
Overview of how a partition containing $N_{el}^{loc}$ can be cast. The different groupings suggest that the maximum number of edges which may require communication is $\propto 2\left(N_{el}^{loc}+1\right)$.

To estimate communication during matrix–vector multiplication, we assume the structure of the mesh decomposition leads to a tree-like graph of communication, arising from recursive bisection. The number of communications will therefore be proportional to $\log_{2}\left(R_{XY}\right)$. Furthermore, we can assume data needs to be exchanged in both directions for each edge of the tree. Message size is far more challenging to estimate, since this is dependent on the size of the boundary between any two partitions. We therefore choose the maximum message size, which can be estimated at $2\left(N_{el}^{loc}+1\right)$, as illustrated in Fig. 4. Here we are also assuming that all partitions are interior to the domain and therefore all boundaries participate in communication. These estimates lead to a prediction for the matrix–vector multiplication communication costs as

$$C_{2}^{E}=2C_{GS}\log_{2}\left(R_{XY}\right)\left[\tau_{L}+2\left(\frac{N_{el}}{R_{Z}R_{XY}}+1\right)\left(P+1\right)\tau_{B}\right]\text{,}$$

where $C_{GS}$ is a constant relating to the implementation of the gather–scatter algorithm.

Combining these contributions gives the cumulative cost of a single iteration of the elliptic solver as

$$T^{E}=\frac{1}{R_{Z}R_{XY}}\cdot \underset{i}{\sum }\overset{E}{\underset{i}{O}}+\underset{j}{\sum }\overset{E}{\underset{j}{C}}\text{.}$$

### 3.5 Incompressible Navier–Stokes model

We now combine the above components of the model to elicit a full model for the incompressible Navier–Stokes algorithm described in Fig. 2. The total parallel execution time for one time-step can be expressed as

(2) $$T^{NS}=a\cdot T^{A}+b\cdot \left(N_{iter}^{P}+3N_{iter}^{H}\right)\cdot T^{E}\text{,}$$

which captures the costs associated with the advection term, Poisson solve for the pressure and the three Helmholtz solves for the velocity components. $N_{iter}^{P}$ and $N_{iter}^{H}$ are the number of iterations of the Poisson and Helmholtz solves, respectively. These will vary depending on the nature of the problem and the choice of preconditioner plays an important role in the efficiency of the iterative solver. The diagonal preconditioner was chosen for modelling simplicity and is not necessarily the most efficient choice. Typical values are $N_{iter}^{P}∼80$ and $N_{iter}^{H}∼10$ for the problems considered in this study. However, these are problem-specific and are largely independent of the parallelisation strategy. The coefficients a and b capture the characteristics of specific hardware and are determined during the calibration process discussed next.

## 4 Results

We consider two prototype turbulent flow problems to quantify the performance of the different parallelisation regimes. These examples highlight the benefits of the hybrid parallelisation approach for increasing parallelism in a scalable way and therefore reducing parallel execution time. Table 1 lists the performance model properties of the two domains considered. Since this study concerns only the parallelisation aspect of these simulations, we consider a fixed discretisation in each case. The discretisation is chosen to be numerically converged for capturing turbulent flow in the given geometry and at the prescribed Reynolds number, based on previously published studies [10], [29], [30].

### 4.1 Test problems

**Fig. 5:**
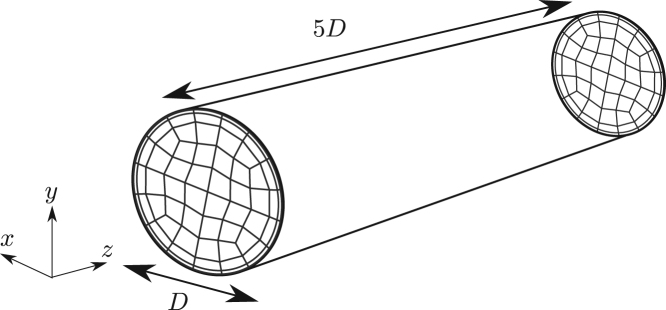
Diagram illustrating the pipe geometry and its discretisation. Spectral/hp elements are used in the cross-plane with a Fourier expansion used in the streamwise direction.

The pipe geometry is illustrated in Fig. 5, where the streamwise direction is geometrically homogeneous. Lengths and velocities are non-dimensionalised by the diameter D and the bulk velocity $u_{\mathrm{bulk}}$, respectively. The length of the pipe is 5D. The flow is driven by a constant body-force of

$$f_{z}=0.5*0.3164/{Re}^{0.25}$$

for Re=3000 to instil a turbulent flow regime. The pipe is discretised using spectral elements in the cross-section of the pipe and a Fourier expansion in the streamwise direction. A total of 64 spectral elements at polynomial order 7 are used in the x–y plane, while 128 modes are used in the Fourier expansion. No-slip boundary conditions are imposed on the wall of the pipe. The time-step used for simulations is Δt=0.002 non-dimensional time units with a second-order IMEX scheme.

**Fig. 6:**
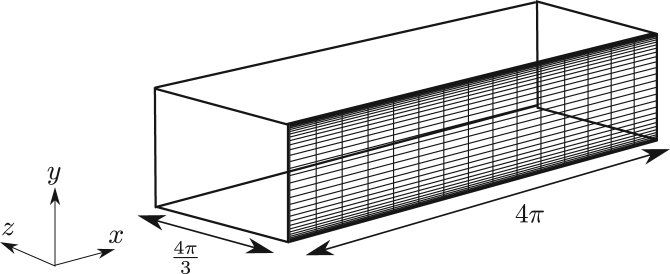
Diagram illustrating the channel geometry and its discretisation. A Fourier expansion is used in the spanwise direction.

For the channel, shown in Fig. 6, lengths are non-dimensionalised by the channel half-height and velocities by $u_{\mathrm{bulk}}$. The length of the channel is 4π, and the spanwise dimension is 4π/3. The flow is driven by a body force of $f_{x}=0.0036$ for Re=3000 and no-slip boundary conditions are imposed on the top and bottom of the channel. The channel was discretised using 64 Fourier modes and 450 spectral elements with a polynomial order of 6. The time-step used for channel flow simulations was 0.0001 with a second-order IMEX scheme.

### 4.2 Hybrid parallelism performance

**Fig. 7:**
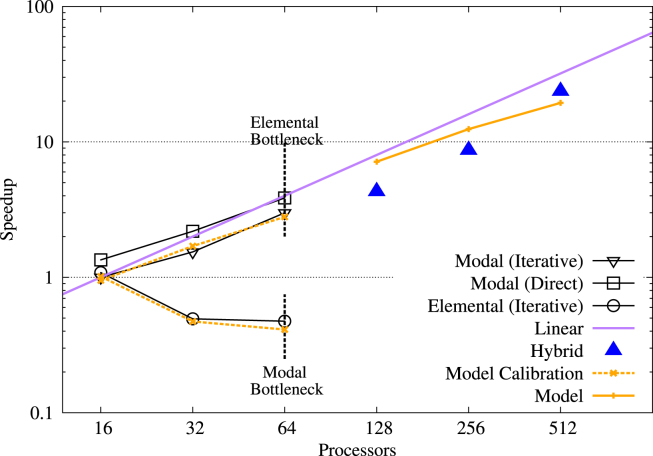
Parallel efficiency of the four parallel approaches for the pipe flow problem on the system detailed in Section  2.4. The vertical dotted black lines indicate the practical limit imposed by the modal and elemental discretisations in isolation. The solid purple line shows ideal efficiency, relative to the 16-core case using modal parallelism with iterative elliptic solver. The dotted orange lines show the model prediction for the cases used for calibration. The solid orange lines show predictions for the hybrid regime. (For interpretation of the references to colour in this figure legend, the reader is referred to the web version of this article.)

The efficiency of the various parallel strategies is assessed through the strong scaling tests for both problems. The results for the pipe are shown in Fig. 7. Modal parallelism (triangle and square symbols) scales well using either direct or iterative elliptic solvers. Elemental parallelism (circle symbols) scales poorly due to the large ratio of communication to computation, since even for only 32 processes, there are only 2 elements per process. The dotted lines indicate theoretical bottlenecks on the number of useable processes due to there being an insufficient number of elements or Fourier modes. In both cases, this limit is 64 processes. However, in the case of a Fourier-dominated discretisation, modal parallelism is clearly preferably over elemental parallelism.

**Fig. 8:**
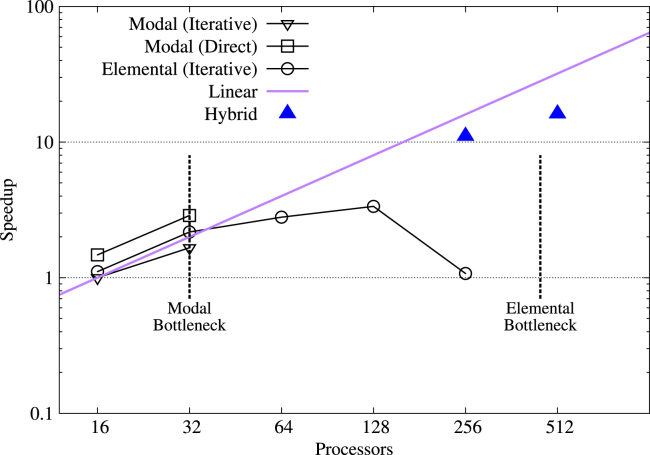
Parallel efficiency of the four parallel approaches for the channel flow problem on the system detailed in Section  2.4. The vertical dotted black lines indicate the practical limit imposed by the modal and elemental discretisations in isolation. The solid purple line shows ideal efficiency, relative to the 16-core case using modal parallelism with iterative elliptic solver.

Fig. 8 shows a comparison of efficiency for the different parallelism strategies in the channel problem. Here the modal bottleneck is reached at 32 cores and the modal approach with direct solver has the greatest performance in this regime. Elemental parallelism is possible up to 450 cores but, above 128 cores, the ratio of computation to communication is low and at 256 cores, the distribution of computation becomes significantly unequal, resulting in poor parallel performance. However, elemental parallelism outperforms modal parallelism when using the iterative solver. This observation is intuitive since only four nodes are used and most of the communication between partitions will be intra-node. Recent versions of the OpenMPI libraries allow processes on the same node to use shared memory, rather than using the network interface to send messages. Within a node latency between processes is therefore low and sending a large number of small messages becomes the most effective method.

Hybrid parallelism extends these limits substantially, enabling simulations up to and beyond 512 processes. The distribution of modal and elemental parallelism will lead to different performance. In Fig. 7, Fig. 8 the solid triangles indicate the minimum execution time achievable using hybrid parallelism for a prescribed total number of cores. Efficiency is less than the ideal case in general, but still reduces runtime significantly as the number of processes increase. In particular, for 512 processes, the performance approaches the ideal case.

**Fig. 9:**
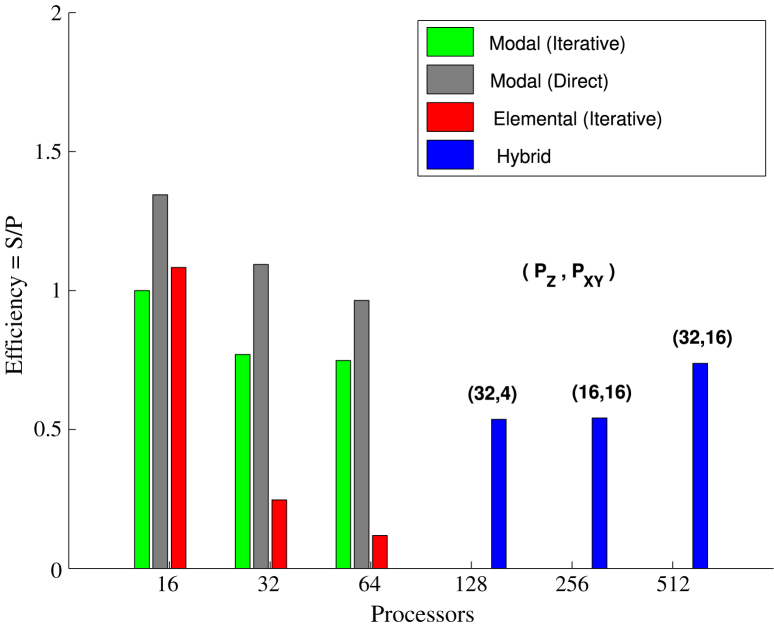
Turbulent pipe flow parallel simulation—efficiency of parallelisation approaches on a cluster of 8-core nodes. The histograms show the efficiency E of different parallel simulations defined as E=S/P where S is the speed-up and P is the total number of processors used for the simulation. The speed-up is based on the 16-core (2 nodes) run using the Modal (iterative) approach.

**Fig. 10:**
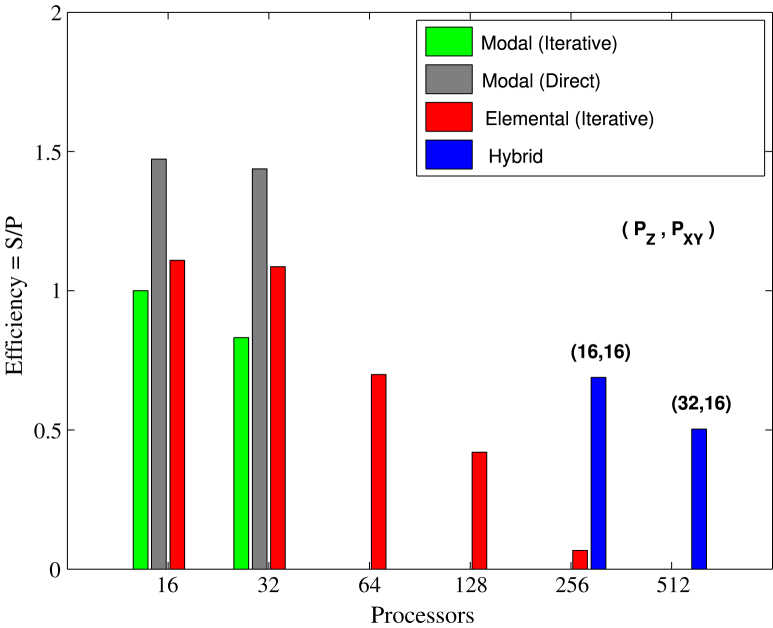
Turbulent channel flow parallel simulation—efficiency of parallelisation approaches on a cluster of 8-core nodes. The histograms show the efficiency E of different parallel simulations defined as E=S/P where S is the speed-up and P is the total number of processors used for the simulation. The speed-up is based on the 16-core (2 nodes) run using the Modal (iterative) approach.

Fig. 9, Fig. 10 show the parallel efficiency of the various parallelisations of the pipe and channel problem, respectively, normalised against the 16-core modal case with iterative solver. Efficiency of both modal and elemental parallelism reduces with increasing core counts, however, the use of the hybrid approach recovers a significant portion of the lost efficiency. For the pipe the combined approach enables 80% of ideal parallel efficiency to be attained on 512 cores, while for the channel there is an order of magnitude increase in efficiency at 256 cores, compared to the elemental approach.

### 4.3 Model calibration

Calibration is the process whereby we identify values for the machine-specific constants a and b in Eq.   (2). To simplify the calibration process, we combine the costs of the elliptic solves and split the timings into those for computation and those for communication as

$$T^{NS}=a_{1}\cdot T_{O}^{A}+a_{2}\cdot T_{C}^{A}+b_{1}\cdot T_{O}^{E}+b_{2}\cdot T_{C}^{E}\text{.}$$

To illustrate the use of the model, six measurements were taken of the time taken to solve the pipe problem using the elemental and Fourier parallel decomposition. The model $T^{NS}$ was implemented in MATLAB and the required coefficients were calculated using a least-squares algorithm as

$$a_{1}=0.45\cdot 10^{-6}\;a_{2}=0.2\;b_{1}=3.15\cdot 10^{-6}$$

and

$$b_{2}=\left\{ \begin{array}{cc} 400\text{,} & \text{if }N_{el}^{plane}/P_{XY}<4\text{,}\\ 10\text{,} & \text{otherwise.} \end{array}\right.$$

The coefficient $b_{2}$ is multi-valued since performance of the elemental parallel decomposition typically degrades sharply when there are fewer than four elements per process, often due to imbalance in the mesh distribution amongst the processes.

### 4.4 Model validation

To quantify the accuracy of predictions using the calibrated performance model in the hybrid regime, we apply the model to the turbulent pipe flow example. Results presented are obtained by averaging 1000 measurements of the timings for each of the components of the time-stepping algorithm. The choice of elliptic solver has a significant impact on performance, and as in the modal parallelism case, the timings for the elliptic solves are measured using both a direct method using LAPACK and an iterative conjugate gradient approach. The four parallelisation types used throughout the remainder of this section are therefore modal (iterative and direct), elemental (iterative) and hybrid (iterative). Strong scaling is performed for the different parallelism strategies and results are normalised by the timings for the 16-core modal approach using the iterative solver.

The model, outlined in Section  3, is calibrated for the turbulent pipe flow example using simulations executed using both modal parallelism and elemental parallelism in isolation. These timings are consequently accurately reproduced by the model, as shown by the dashed orange lines in Fig. 7. The solid orange line in the same figure shows predicted runtimes using the performance model in the hybrid regime. Good agreement is observed and the model correctly identifies the best performing hybrid case, timings of which are shown by the blue triangles.

## 5 Discussion

In this paper we presented a technique to parallelise a 3D incompressible Navier–Stokes algorithm discretised using a 2D spectral/*hp* element mesh coupled with a Fourier expansion in a third geometrically homogeneous direction. The implementation enables a flexible mixture of both elemental parallelism and modal parallelism. We have illustrated the hybrid parallelism technique on two prototype problems: turbulent flow in an axisymmetric pipe and turbulent flow in a channel. Both problems enjoy increased parallelism through the approach and consequently improved runtimes and greater energy efficiency. The optimal weighting of these strategies can be systematically chosen through the construction of a performance model, calibrated to a specific system through measurements of the two parallelism approaches independently. This enables rapid selection of the highest-performing combination of the strategies without costly trial-and-error testing.

In the modern HPC environment, where energy is of increasing concern, selecting the optimal implementation to maximise performance is becoming increasingly important. Although experience and intuition can generally suggest the most suitable parallelisation approach for a specific problem, the decision is in general highly challenging, particularly when moving between HPC systems or when tackling problems on a range of different domains with the same algorithm. Even from a purely theoretical perspective, it can be appreciated that a single parallel approach cannot be optimal in all situations and this has been confirmed through numerical experimentation.

The number of degrees of freedom in the xy-plane and the number of Fourier modes are the first indicators of which technique is more appropriate. We recognise that problems with a high number of modes compared to the number of elements per plane appear to benefit from the modal parallelisation approach. On the other hand, a domain discretisation with a larger number of elements than Fourier modes generally benefits from an elemental decomposition technique. However, the relative performance of different approaches cannot be determined entirely through operation counts. Accounting for specific hardware characteristics and the latency and bandwidth available on the communication pattern is essential to accurately predict the optimal strategy. Parallelisation approaches requiring a large number of messages, such as the mesh-decomposition parallelisation, can suffer from poor performance if the interconnect latency is high. These types of parallel techniques are therefore efficient on shared memory machines or low-latency interconnects.

In general we wish to be able to tackle a range of problems where those quantities can vary, potentially reaching extreme values. It is therefore clearly beneficial to have both parallelisation strategies available within a single codebase and this study illustrates the advantages of being able to combine them in a flexible manner to achieve lower runtimes on a fixed number of processors. A further benefit is the extension of strong scalability possible through the use of the hybrid parallel implementation. The potential of new machines are often exploited by investigating even larger problems than previously explored and in finer detail, or using larger Reynolds numbers, and to capitalise on weak scalability. Good weak scalability generally follows from good strong scalability and by increasing an algorithms strong scalability, simulations can be run faster and on larger machines.

It should be noted that not all the hybrid parallel approaches we tested provided good performance. Depending on the mesh topology, partitioning and the number of Fourier modes on each processor, some strategies may not perform efficiently. We note, for example, that the optimal 128-core hybrid parallel case for the turbulent pipe in Fig. 7 has a similar runtime to the modal parallelism using the direct solver with only 64 cores. Conversely, we showed that certain choices may result in reduced computational time without increasing the number of processors. This is the case of the modal approach when using a direct solver, which generally performs as well as an elemental parallelisation method with twice the number of CPUs. Minimising the energy consumption when running a simulation is a point of interest in current high performance computing research. Implementation flexibility plays an important role in addressing these goals.

In terms of limitations, we have disregarded some pieces of the algorithm in order to focus on the two main routines, namely the advection and the elliptic operators. This is a typical approach when creating a scalability model [20], although it may introduce some errors. The calibration has been carried out by monitoring the solution time on the SGI Altix ICE 8200 EX system, therefore the coefficients presented here must be considered specific for that machine. Finally, the model, and therefore the results presented in this paper, are specific to *Nektar++*. However, it still provides valuable insight and can suggest overall guidelines on typical choices of parallelisation strategy on large HPC resources.

## Figures and Tables

**Table 1:** Turbulent test cases discretisation features.

Test case	P	$N_{el}^{plane}$	$N_{Z}$	$N_{el}$	$N_{XY}$	$N_{TOT}$
Pipe	7	64	128	8 192	5 184	663 552
Channel	6	450	64	28 800	28 800	1843 200
